# Unraveling Factors Shaping the Acceptance and Non-acceptance of Non-scalpel Vasectomy in Rural Central India: A Cross-Sectional Study

**DOI:** 10.7759/cureus.53311

**Published:** 2024-01-31

**Authors:** Sarita K Sharma, Gopal Patil, Prashant Ghunkikar, Pragati G Rathod, Komal Dhumal

**Affiliations:** 1 Community Medicine, Government Medical College (GMC) Nagpur, Nagpur, IND; 2 Leprosy, Maharashtra Public Health Services, Yavatmal, IND; 3 Community Medicine, Bharti Vidyapeeth Medical College and Hospital, Sangli, IND

**Keywords:** cross-sectional study, india, socio-demographic factors, contraceptive acceptance, family planning, non-scalpel vasectomy

## Abstract

Background

India, with a population exceeding 1.43 billion, faces significant demographic challenges, necessitating effective family planning measures. Non-scalpel vasectomy (NSV) is a less complex and cost-effective male contraceptive, yet its acceptance remains low, especially compared to female sterilization. Understanding the determinants of NSV acceptance is crucial for informed decisions on family planning methods.

Methods

This cross-sectional study investigated NSV acceptance among males who underwent non-scalpel vasectomy (acceptors of NSV) and spouses of women who underwent tubectomy (non-acceptors of NSV). The study was conducted among 116 NSV acceptors and 116 non-acceptors from rural Central India. Data were collected over six months, employing a pre-designed questionnaire covering socio-demographic details, reasons for acceptance/non-acceptance, and information sources for the same. Statistical analysis was done utilizing Epi Info 7.2.6 (Centers for Disease Control and Prevention [CDC], Atlanta, GA), employing descriptive statistics and tests of association.

Results

Significant associations were found between NSV acceptance (p<0.05) and the age and education of study subjects, age of wife, duration since marriage, and total number of children. Incentives also played a significant role (p=0.014). Opposition to NSV, mainly from wives, was a key factor for non-acceptance (38%). Reasons for acceptance included a previous cesarean section (40.52%), the simplicity of the NSV procedure (26.72%), and the wife's illness (23.28%). Non-acceptance reasons comprised family/friend opposition (38%), lack of awareness/ignorance (25.00%), and fear of surgery (23.28%). Study subjects perceived community reluctance to NSV as mainly due to misbeliefs (30.17%), fear of surgery (27.58%), and illiteracy (26.29%).

Conclusion

The study highlights socio-demographic factors influencing NSV acceptance and identifies key reasons for acceptance/non-acceptance. Community-based interventions, increased advertisement, and health provider counseling were suggested for enhancing acceptance. Despite challenges, participant satisfaction with NSV was high. These findings contribute to understanding the complex dynamics surrounding NSV acceptance in rural Central India, informing future family planning strategies.

## Introduction

India is the most populous country globally, constituting approximately 17.76% of the world's population. According to UN estimates, India's population has exceeded 1.43 billion, accompanied by a growth rate of 0.81%, leading to India surpassing China and securing the position of the world's most populous nation [1]. India contributes a substantial 20% to the total global births, and this demographic enormity places significant strain on its resources [2].

In response to the demographic challenge, India initiated the National Family Welfare Program in 1951, aiming to curtail the birth rate and stabilize the population in alignment with the national economic needs [3]. In a societal framework characterized by male dominance, the active involvement of men in family planning emerges as an imperative for the efficacy of such programs [4].

Non-scalpel vasectomy (NSV) represents a highly effective contraceptive modality for males. Despite its distinct advantages, including a shorter, safer, and less complex procedure with minimal complications, swifter recovery, and cost-effectiveness, the acceptance of vasectomy remains relatively low in developing countries, particularly in India. The National Family Health Survey V data underscores a stark contrast, with merely 0.3% of couples opting for male sterilization in comparison to 37.9% adopting female sterilization [5]. Moreover, the acceptance of NSV in India has remained unchanged from the NFHS IV to the NFHS V, indicating persistent challenges despite extensive efforts, including national health programs [5]. Although awareness of NSV is notable, its translation into actual utilization is limited, with female sterilization maintaining a dominant position in the family planning landscape [6].

Scientific investigations reveal that both men and women harbor reservations toward vasectomy, citing concerns about its efficacy and apprehensions related to potential physical weakness [7,8]. Such concerns, rooted in historical experiences, present formidable barriers to the adoption of NSV. In contrast, female sterilization garners widespread acceptance, with societal norms potentially influencing the perception that weakness in women is more tolerable given traditional gender roles [4].

Factors contributing to the reluctance toward NSV encompass inadequate knowledge, societal influences, low literacy rates, disproportionate emphasis on female sterilization, male ego, and misconceptions regarding its impact on libido [6]. Notably, family planning initiatives and clinics predominantly focus on women, often staffed by female personnel.

Given the imperative for informed and voluntary acceptance of NSV, a comprehensive understanding of the interplay between social and medical determinants is essential. This cross-sectional study, conducted among males undergoing NSV and spouses of tubectomy acceptors, seeks to elucidate the nuanced determinants of acceptance of NSV, particularly within the context of limited data available from central India.

## Materials and methods

Study design, setting, population, and duration

This cross-sectional study was carried out in rural central India to investigate the factors determining the acceptance and non-acceptance of NSV. The study population comprised males who had undergone the NSV procedure (i.e., acceptors of NSV) and the spouses of females who had chosen tubectomy as a means of permanent sterilization (i.e., non-acceptors of NSV). The study was conducted over six months, from December 15, 2022 to June 14, 2023.

Ethical considerations

Before commencing the study, the necessary approvals were obtained from the institutional ethics committee. In addition, permission to conduct the research was sought from the District Health Officer, who was provided with a comprehensive overview of the study's nature and objectives.

Sample size and sampling

The study's projected sample size was determined based on the predominant factor influencing NSV acceptance, as identified in a prior cross-sectional study conducted in Mangalore by Shettian and Ajila [9] in 2018, where the observed proportion was 27.8%. Calculations were performed with a relative precision of 9% and a confidence level of 95%, resulting in an estimated sample size of 95 participants. Accounting for a non-response rate of 10%, the final actual sample size for both groups comprised 115 participants. However, the actual sample size included 116 participants in both groups, i.e., acceptors and non-acceptors.

From the 49 primary health centers in Nagpur district, seven primary health centers were selected using a random sampling method (simple random sampling by lottery). Each primary health center was visited on the day of the scheduled Family Planning Camp, with specific dates previously established for each primary health center.

Data collection

Written, informed consent was obtained from all study subjects after providing them with a clear and comprehensive explanation of the study's purpose. The confidentiality of the study participants was assured and diligently maintained throughout the study.

Data were collected using a pre-designed and pre-tested questionnaire administered through interview techniques. This questionnaire covered a range of information, including socio-demographic details, reasons for the acceptance or non-acceptance of NSV, and the sources of information regarding NSV. Interviews were conducted in a separate, private room provided by the medical officer of the PHC to ensure a confidential and conducive environment for participants.

A pilot study was conducted on 20 subjects (10 in each group) as a preliminary step before commencing the main study. These were excluded from the final analysis. This pilot study served the purpose of assessing the feasibility of the research and testing the questionnaire. Necessary modifications and adjustments were made based on the insights and feedback obtained during the pilot study.

Statistical analysis

Data entry and analysis were done using the statistical software Epi Info 7.2.6 (Centers for Disease Control and Prevention [CDC], Atlanta, GA). Descriptive statistics (percentage, mean, standard deviation, range) were used to summarize baseline characteristics of the study subjects. The correlation between two categorical variables was analyzed using the chi-square and Fisher exact tests and a 95% confidence interval (CI). A P-value <0.05 was considered to be statistically significant.

## Results

In total, the study encompassed 116 acceptors and the same number of non-acceptors of NSV. Table 1 presents the distribution of study subjects across various sociodemographic factors and their influence on NSV acceptance. The majority of participants were below 34 years of age, with 60 (51.37%) being acceptors and 72 (62.10%) non-acceptors. Acceptors had a mean age of 33.74 (± 4.82) years, while non-acceptors had a mean age of 32.90 (+3.92) years. Of the individuals who accepted, 77 (66.42%) had an education level above middle school, whereas the corresponding figure for non-acceptors was 47 (40.52%). The religious affiliation of the majority in both groups was Hindu, with 88 (75.90%) acceptors and 92 (79.31%) non-acceptors. Chi-square tests revealed significant associations between the age of the wife (p=0.001), duration since marriage (p=0.02), and total number of children (p=0.001) with the acceptance of NSV.

**Table 1:** Sociodemographic factors and their role in NSV acceptance NSV: non-scalpel vasectomy *Statistically significant **As per the chi-square test value Acceptors - those who underwent NSV Non-acceptors - spouses of women who underwent tubectomy

Variable		NO	Acceptors		Non-acceptors		P-value**
			N	%	N	%	
Age	≤34 years	132	60	4546	72	54.54	0.1
	≥34 years	100	56	56.00	44	44.00	
Education	Up to middle school completion	116	39	33.62	77	66.38	0.34
	Above middle school completion	116	47	40.51	69	59.49	
Occupation	Unskilled	134	69	51.50	65	48.50	0.5
	Semi-skilled and above	98	47	48.95	51	52.05	
Religion	Hindu	185	88	47.57	97	52.43	0.4
	Others	52	28	53.84	24	46.16	
Age of wife	<29 years	159	67	42.13	92	57.87	0.001*
	>29 years	73	49	67.12	24	32.88	
Education of wife	Up to middle school completion	87	40	45.97	47	54.03	0.2
	Above middle school completion	139	76	54.67	63	45.33	
Occupation of wife	Homemaker	142	70	49.29	72	50.71	0.78
	Others	90	46	51.11	44	48.89	
Socioeconomic status	I and II	27	18	66.67	09	33.33	0.06
	III to V	205	98	47.80	107	52.20	
Type of family	Nuclear	165	77	46.67	88	53.33	0.1
	Joint and three-generation	67	39	58.20	28	41.80	
Duration since marriage	<5 years	203	96	47.29	107	52.71	0.02*
	≥5 years	29	20	68.96	09	31.04	
Total no of children	≤3	184	102	55.43	82	44.57	0.001*
	>3	48	14	29.16	34	70.84	

**Figure 1:**
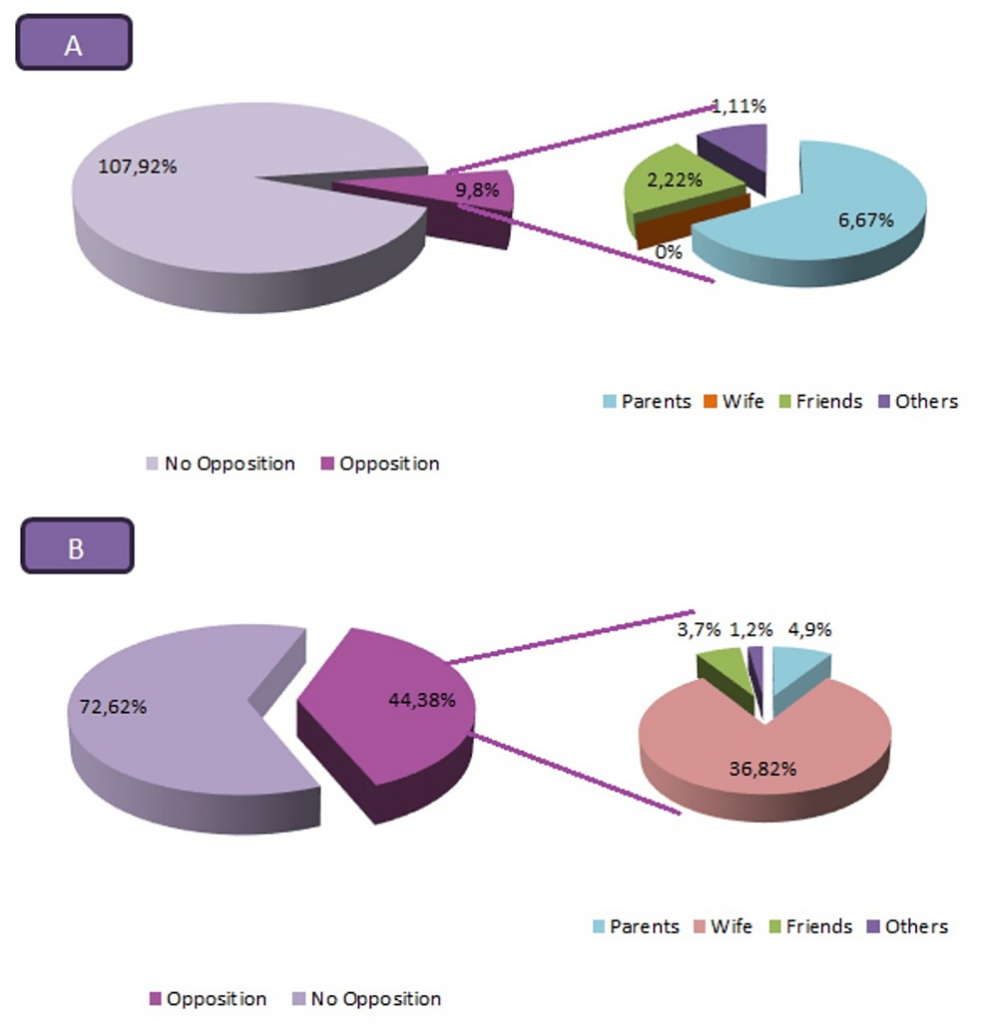
Opposition to non-scalpel vasectomy from family and friends NSV: non-scalpel vasectomy; A: acceptors of NSV; B: non-acceptors of NSV

Figure 1 illustrates the distribution of study participants based on the opposition to NSV from family and friends. Among those who opted for the procedure, 8% acknowledged facing opposition. In contrast, among the non-acceptors, approximately 38% revealed encountering opposition from family members and friends, with wives being the primary source of opposition, accounting for 82% of non-acceptors.

**Figure 2:**
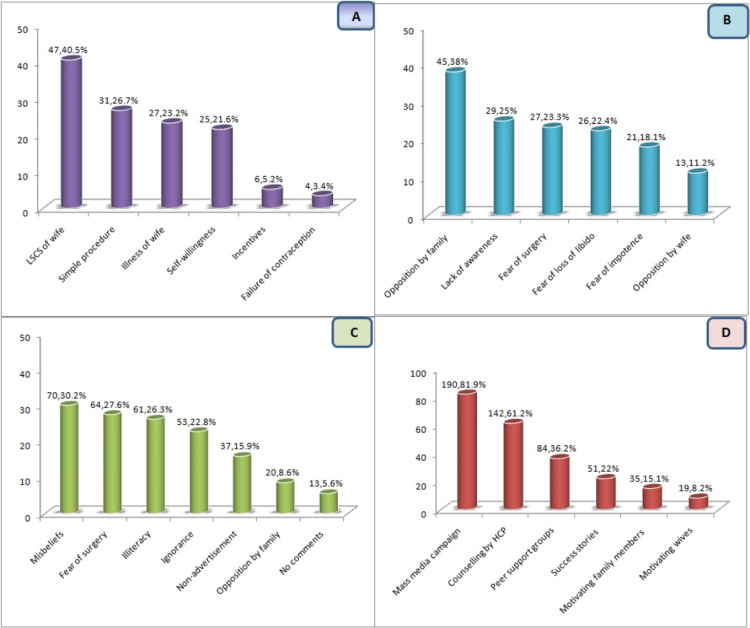
Factors influencing acceptance and non-acceptance of NSV at individual, and community levels, and suggestions for improvement NSV: non-scalpel vasectomy Y-axis in all figures: number of study subjects A: reasons for acceptance of NSV (N=116); B: reasons for non-acceptance of NSV (N=116); C: perceptions leading to poor acceptance of NSV (N=232); D: suggestions to improve acceptance of NSV (N=232)

The reasons for acceptance of NSV are depicted in Figure 2A. A significant proportion of the study participants, comprising 40.52%, cited the previous lower segment cesarean section (LSCS) of their wives as the primary motivating factor for opting for NSV. Other reported reasons included the perception of the procedure being simple and easy (26.72%), the illness of the wife (23.28%), and personal willingness (21.55%). Additionally, a small percentage (5.17%) of participants chose NSV due to incentives, while a minority (3.44%) opted for it as a result of the failure of a previously used method.

Figure 2B outlines the reasons for the non-acceptance of NSV. The predominant reason, cited by 38% of participants, was opposition from family and friends. Other reasons included lack of awareness/ignorance (25.00%), fear of surgery (23.28%), fear of loss of libido (22.43%), and fear of impotence (18.10%).

Reasons for the poor acceptance of NSV in the community as perceived by the study subjects are seen in Figure 2C. A total of 05.60% of participants refrained from providing comments. The most prevalent reasons for the community's reluctance towards NSV, as perceived by the study participants, included misbeliefs (30.17%), fear of surgery (27.58%), and illiteracy (26.29%). Additionally, some study subjects attributed poor acceptance to non-advertisement (08.62%), while opposition by family members was mentioned by 05.60%.

Figure 2D illustrates the methods proposed by study subjects to enhance the acceptance of non-scalpel vasectomy. The most frequently suggested approach was increased advertisement through mass media, supported by 81.89% of participants, followed by counseling by health care providers at 61.2%. Motivating family members was recommended by 15.08% of respondents, while utilizing the success stories of non-scalpel vasectomy acceptors for motivation was endorsed by 21.98% of study subjects.

**Figure 3:**
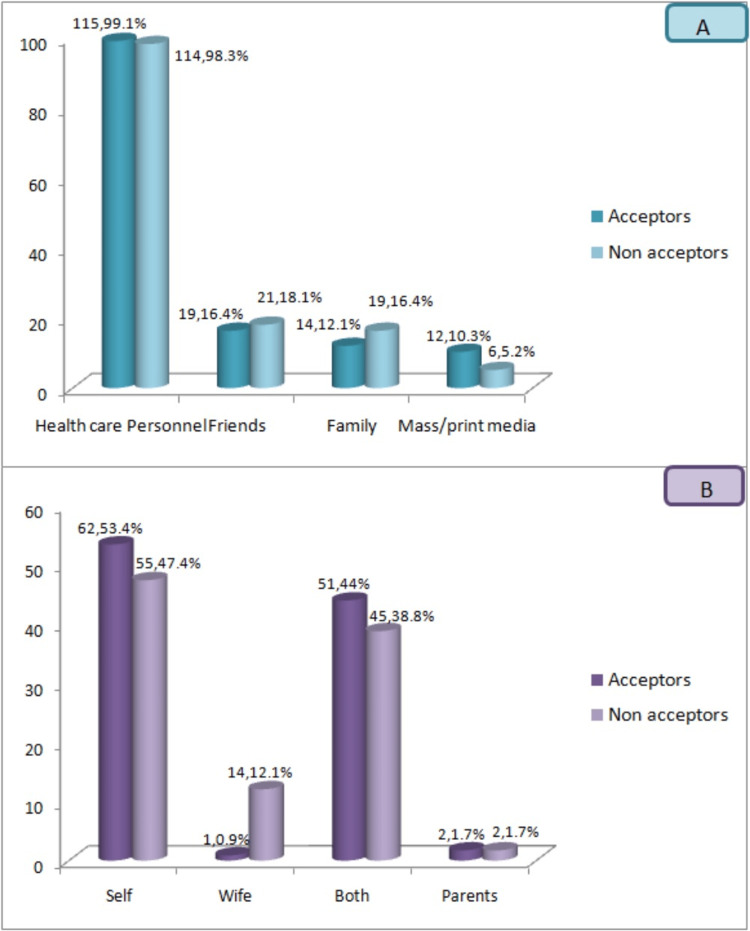
Source of information regarding non-scalpel vasectomy A: source of information for non-scalpel vasectomy; B: decision-maker for permanent sterilization Y-axis in all figures: number of study subjects

The source of information regarding NSV is illustrated in Figure 3A. The majority of both acceptors 115 (99.14%) and non-acceptors 114 (98.28%) indicated that the primary source of information was healthcare personnel, either from the public or private healthcare sector. Additionally, significant sources of information included mass/print media, as well as family and friends.

The distribution of study subjects based on the decision-maker for permanent sterilization is depicted in Figure 3B. In most of the cases, the study subjects made the decision themselves, accounting for 62 (53.45%) acceptors and 55 (47.41%) non-acceptors. Alternatively, the decision was a joint one involving both the husband and wife, constituting 51 (43.97%) acceptors and 45 (38.79%) non-acceptors.

**Figure 4:**
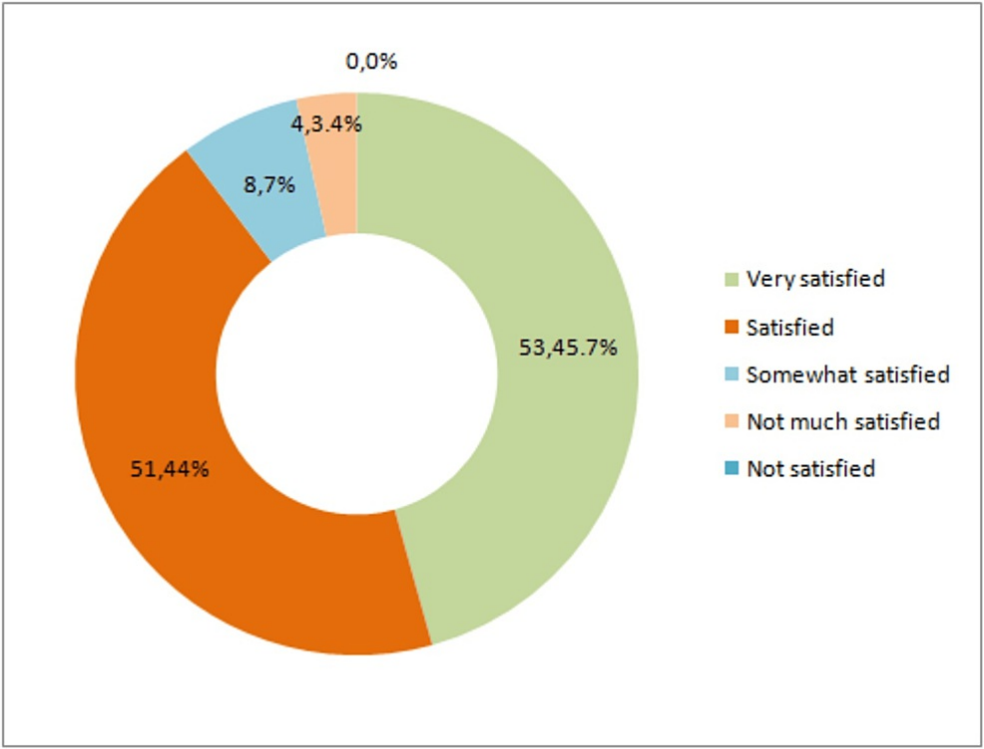
Level of satisfaction for non-scalpel vasectomy

The findings regarding participant satisfaction with NSV reveal a predominantly positive sentiment, as seen in Figure 4. A substantial proportion of participants expressed being "very satisfied," accounting for 45.69% of the responses. Additionally, a significant number reported being "satisfied" or "somewhat satisfied," constituting 43.97% and 6.90% of the participants, respectively. Interestingly, there were no participants who expressed being "not satisfied" with non-scalpel vasectomy, suggesting a high overall satisfaction rate among the study participants.

## Discussion

In recent years, there has been a growing emphasis on male partners taking on increased responsibility in household and family matters. To encourage greater male participation in family planning, it is essential to offer a wide range of safe and effective male contraceptive options. NSV is one such method. Despite males being primary decision-makers in reproductive matters, their acceptance of NSV as a family planning method has been less than satisfactory thus far [10].

Compared to female surgical sterilization, vasectomy is notably safer, easier, and entails a quicker recovery. The procedure is less time-consuming, causes less pain, and can be performed in an outpatient setting with a local anesthetic, eliminating the need for an incision. The chances of bleeding are reduced, recovery is expedited, and the failure rate is lower [11].

While awareness of non-scalpel vasectomy is widespread, its utilization is minimal, with female sterilization remaining the predominant method of choice. Despite efforts made through national health programs, which include the provision of health manpower, training, political commitment, and advancements in surgical techniques, the low acceptance of male sterilization persists in comparison to the significant number of women opting for sterilization. This discrepancy may be attributed to factors such as inadequate knowledge, social influences, illiteracy, disproportionate preference for female sterilization, male ego, and misconceptions about its potential impact on libido. Most study subjects fell within the age group of 30-39 years, with the mean age for acceptors and non-acceptors being 33.74 ± 4.82 years and 32.90 ± 3.92 years, respectively. These trends align with findings from various studies conducted across the globe by different authors [12-15]. The majority of the study subjects in the present study were Hindu. Strikingly, none of the acceptors of NSV in our study were Muslims. This observation mirrors a broader trend suggesting that vasectomy may not be widely adopted among Muslims and followers of other religions, as indicated by studies such as Mahapatra et al. [15].

Education is an important determinant of acceptance of NSV, but in the present study, no significant difference was observed between the educational level of acceptors and non-acceptors of NSV. These findings were in accordance with the observations of other authors [10,12,16].

As per the findings of the present study, wives of NSV acceptors were comparatively older than those of non-acceptors, and this difference was statistically significant. Presumably, older couples may be more open to newer family planning methods like NSV due to changing societal norms. They may prioritize health considerations, and NSV, being less invasive and having a quicker recovery, may be perceived as a suitable option. Similar observations were also reported by Zafer et al. [12].

More than half of the study subjects, i.e., 56.03% of acceptors and 59.49% of non-acceptors, were unskilled workers. These findings contradict common misconceptions that vasectomy is not suitable for individuals engaged in heavy labor. Similar observations were also reported by Saoji et al. [14] and Sivagnanam et al. [17]. This challenges the notion that vasectomy is incompatible with strenuous occupations, as evidenced by the engagement of vasectomized individuals in physically demanding work.

In joint family setups, senior members often influence decisions, serving as both motivators and demotivators. But in the present study, 66.38% of acceptors and 75.86% of non-acceptors belonged to nuclear families. Similar observations were reported by other authors [10,16].

The acceptability of NSV demonstrates considerable variation based on the duration of the marriage. The majority of the study subjects, both acceptors (69.93%) and non-acceptors (78.45%) had been married for five to nine years. These findings are consistent with those of other authors [10,12,13,18]. This highlights the influence of the duration of marriage on the acceptability of NSV, underscoring the importance of considering marital longevity in understanding and addressing attitudes toward this family planning method.

It was observed in the present study that families with fewer than three children had a greater acceptance of NSV as compared to others. This can be explained based on the fact that families with fewer children may have a higher level of awareness and education about family planning options, including NSV. Increased knowledge may contribute to a more positive attitude toward NSV.

The current study revealed a significantly higher acceptance of NSV among families with fewer than three children compared to others. This trend can be elucidated by the notion that families with fewer children tend to exhibit heightened awareness and education regarding various family planning options, including NSV. The increased knowledge levels within these families likely play a pivotal role in fostering a more positive attitude toward NSV as a viable family planning method.

The observation that wives of both acceptors and non-acceptors tend to fall within a similar age range may indicate that the age of the wife alone might not be a determining factor influencing the acceptance or rejection of NSV. Other social, cultural, or personal factors could play a more significant role in decision-making regarding family planning methods. Further exploration and analysis of these factors may provide insights into the nuanced dynamics affecting NSV acceptance within this demographic.

The present study highlights that a meager percentage of NSV acceptors faced opposition from family and friends, indicating generally positive social support. In contrast, one-third of the non-acceptors encountered opposition, predominantly from wives. This underscores the significant impact of spousal influence on NSV decisions.

Opposition from family and friends, ignorance, and fear of loss of libido were the common reasons cited for non-acceptance of NSV in the present study. Similar reasons were also reported in studies carried out by Ayele et al. [11].

The most common approach suggested by participants for improving NSV acceptance was increased advertisement through mass media, followed by counseling by health care providers, motivating family members, and utilizing success stories of non-scalpel vasectomy acceptors. The need for rigorous mass media campaigns to highlight the advantages of NSV and its availability was also endorsed by Khokhar et al. [13].

The primary source of information for NSV was healthcare personnel and the media. Similar findings were also reported by various authors [19-21]. In most of the cases, the study subjects made the decision themselves; alternatively, the decision was a joint one involving both the husband and wife.

The present study is subject to the inherent limitations associated with a cross-sectional research design. Given the limited number of NSV acceptors in the population, their selection was based on availability, specifically those attending family planning camps at selected primary health centers; hence, the study findings may not be universally applicable to the entire male population.

## Conclusions

The study highlights a positive trend toward male partners taking increased responsibility in family planning decisions, aligning with the contemporary emphasis on shared roles and responsibilities within households. The higher acceptance of NSV among families with fewer than three children indicates a need for tailored family planning interventions based on family size considerations. Age-related patterns reveal that older couples, particularly those with older wives, show a greater acceptance of NSV. This suggests a potential correlation between age, maturity, and family planning decisions, emphasizing the importance of considering the life stage of couples. Addressing opposition from family and friends, especially from spouses, is crucial for enhancing NSV acceptance.

The observed discrepancy in NSV acceptance between acceptors and non-acceptors highlights the necessity for targeted educational campaigns. Dispelling myths, addressing concerns, and promoting understanding could contribute to a more supportive environment for NSV adoption. Acknowledging the influence of cultural, educational, and socioeconomic factors in family planning decisions is important. Interventions should be multifaceted, aiming to bridge knowledge gaps and dispel misconceptions within diverse demographic groups. The study underscores the importance of healthcare providers and community leaders in facilitating open conversations about family planning. Their role in addressing concerns and promoting the benefits of NSV can contribute to a positive shift in societal attitudes. Future research and interventions should continue exploring and addressing the multifaceted factors influencing NSV acceptance, considering both individual and social perspectives, to develop more effective family planning strategies.
